# An ecological time-series study of heat-related mortality in three European cities

**DOI:** 10.1186/1476-069X-7-5

**Published:** 2008-01-28

**Authors:** Ai Ishigami, Shakoor Hajat, R Sari Kovats, Luigi Bisanti, Magda Rognoni, Antonio Russo, Anna Paldy

**Affiliations:** 1 Public & Environmental Health Research Unit, London School of Hygiene & Tropical Medicine, Kappel Street, London, WC1E 7HT, UK; 2 Azienda Sanitaria Locale della Città di Milano, Servizio di Epidemiologia, Corso Italia, 19, 20122 Milano, Italy; 3 József Fodor National Centre of Public Health, National Institute of Environmental Health, Department of Biological Monitorino, Gyali ut 2-6, PO.Box. 64, 1097 Budapest, Hungary

## Abstract

**Background:**

Europe has experienced warmer summers in the past two decades and there is a need to describe the determinants of heat-related mortality to better inform public health activities during hot weather. We investigated the effect of high temperatures on daily mortality in three cities in Europe (Budapest, London, and Milan), using a standard approach.

**Methods:**

An ecological time-series study of daily mortality was conducted in three cities using Poisson generalized linear models allowing for over-dispersion. Secular trends in mortality and seasonal confounding factors were controlled for using cubic smoothing splines of time. Heat exposure was modelled using average values of the temperature measure on the same day as death (lag 0) and the day before (lag 1). The heat effect was quantified assuming a linear increase in risk above a cut-point for each city. Socio-economic status indicators and census data were linked with mortality data for stratified analyses.

**Results:**

The risk of heat-related death increased with age, and females had a greater risk than males in age groups ≥65 years in London and Milan. The relative risks of mortality (per °C) above the heat cut-point by gender and age were: (i) Male 1.10 (95%CI: 1.07–1.12) and Female 1.07 (1.05–1.10) for 75–84 years, (ii) M 1.10 (1.06–1.14) and F 1.08 (1.06–1.11) for ≥85 years in Budapest (≥24°C); (i) M 1.03 (1.01–1.04) and F 1.07 (1.05–1.09), (ii) M 1.05 (1.03–1.07) and F 1.08 (1.07–1.10) in London (≥20°C); and (i) M 1.08 (1.03–1.14) and F 1.20 (1.15–1.26), (ii) M 1.18 (1.11–1.26) and F 1.19 (1.15–1.24) in Milan (≥26°C). Mortality from external causes increases at higher temperatures as well as that from respiratory and cardiovascular disease. There was no clear evidence of effect modification by socio-economic status in either Budapest or London, but there was a seemingly higher risk for affluent non-elderly adults in Milan.

**Conclusion:**

We found broadly consistent determinants (age, gender, and cause of death) of heat related mortality in three European cities using a standard approach. Our results are consistent with previous evidence for individual determinants, and also confirm the lack of a strong socio-economic gradient in heat health effects currently in Europe.

## Background

Europe has warmed by 0.3°C per decade since the 1970s [1] and most countries have experienced an increase in the number of heat episodes in the past two decades [2]. As public health measures are developed to reduce the impacts of heat-waves and hot weather, there is a need to better describe the environmental and social determinants of heat-related mortality.

In addition to physiological and clinical studies of heat stress, there is a growing literature of epidemiological studies that look at risk factors for heat-related mortality. These studies indicate that the elderly are at highest risk of heat-related mortality. However, many of these studies have used very broad age groups and do not sufficiently adjust for age when looking at gender or other subgroups, and therefore estimates are potentially subject to residual confounding. A multi-city case-crossover analysis in four Italian cities found the risk of heat related mortality was higher in women compared to men[3], after adjusting for age, but other studies have shown no differences by gender. Few studies have investigated the effect of socio-economic status on heat mortality in Europe. Excess mortality in Rome during the heat-wave of 2003 was lower in persons with the highest level of education [4], however other studies in Barcelona [5], Paris [6] and the UK [7] have shown little difference in impacts between high and low income groups.

We investigated the effect of heat on daily mortality in three European cities. The three cities selected were partners in the EUROHEAT project on improving public health responses to extreme weather [8]: Budapest (Hungary), London (UK), and Milan (Italy) and represent a range of climates across Europe.

Budapest has a continental climate, with relatively high summer temperatures and recurrent heat-waves are a problem [9]. Exposure to air pollutants is relatively high compared to other countries in Europe [10,11]. London has a maritime climate, and a well described heat island which means that summer night time temperatures can be 1–3°C higher than the surrounding areas [12]. Air pollution in London is relatively modest, although particulate matter and ozone levels were elevated during the 2003 heat-wave event [13]. Recent research has shown London is more sensitive to heat-related mortality than other regions or urban areas in England and Wales [7]. Milan and London were both severely affected by the 2003 heat-wave. Approximately 560 excess deaths (an increase of 23% in total mortality) were observed in Milan [4,14] and 616 deaths (42%) in Greater London [15] during the heat event. Temperatures in Budapest were not much influenced by the heat-wave which predominantly affected western Europe. In all three cities, heat health warning systems were implemented after the 2003 event [9,16,17].

We investigated a range of risk factors of heat-related death using routine mortality data linked to small area indicators of environment and social status. This comparative study uses a standard time-series approach to identify the general determinants of heat-related mortality in European settings.

## Methods

### Mortality data

Mortality data were supplied by the Central Statistical Office in Budapest, the Office for National Statistics (ONS) in the UK, and the Local Health Authority in Milan. Daily counts of deaths from all-cause, cardiovascular disease (*International Classification of Diseases*, ICD-9 390.0–459.9 for deaths before 2001; ICD-10 I for deaths after 2001), respiratory disease (ICD-9 460.0–519.9; ICD-10 J), and external disease (ICD-9 900.0–999.9; ICD-10 S,T,V,W,X,Y,Z) were obtained for each city. We created mortality series for the following narrow age bands (0–14, 15–64, 65–74, 75–84, ≥85 years).

Linkage of mortality to deprivation indices was based on the best data available for each city. Information on income was derived from the published 2001 census in each city. In London, we computed population-weighted average score of the Multiple Deprivation Index (MDI) by Census Area Statistic ward. For Milan, we used the annual income (median) of residents by census tract after linking data from the Tax Register with the Milan Population Registry. Small area statistics were not available in Budapest so census data were linked at district-level. We used the proportion of the population who completed at least secondary school education as the indicator for socio-economic status. Other factors available from the census, such as dwelling type and proportion of elderly residents living alone, were also linked to investigate their effects on heat-related mortality.

### Weather and pollution data

Series of daily mean temperature (°C) were generated for each city (Table 1). Air pollution may confound the effects of high temperature on mortality, and so daily ambient levels of PM_10 _(μg/m^3^) (total suspended particles (TSP) in Budapest), and ozone (μg/m^3^) were obtained for each city. For Budapest and London, reference monitoring stations were selected based on criteria of geography and least missing data. The Milan data (from four monitoring sites) were provided by the Regional Environmental Protection Agency.

**Table 1:** Characteristics of study cities

	Budapest	London	Milan
Study period	1993–2001	1993–2003	1999–2004
Population	1698106	7517700	1305808
Latitude	47°30'N	51°30'N	45°27'N
Average summer (June-Aug) temperature, °C	21.3	18.1	23.0
Heat cut-point. 95th percentile daily mean temperature, °C	24.4	20.4	26.3
Particulate concentrations, μg/m^3^ Mean (5th to 95th percentile)	TSP: 57.0 (26.0 – 104.5)	PM_10_: 32.9 (18.5–60.5)	PM_10_: 58 (19–137)
8 hr Ozone concentration, μg/m^3^ Mean (5th to 95th percentile)	73.3 (26.0 – 132.5)	21.8 (5.5 – 46.0)	74 (9 – 173)
Daily number deaths Mean (5th to 95th percentile)	75 (56 – 97)	171 (134 – 223)	30 (19 – 43)
Age at death (years), %			
0–14	0.8	1.4	1
15–64	26.2	18.3	14
65–74	23.6	19.6	20
75–84	29.3	31.6	31
85+	20.1	28.3	34
Cause of death, %			
CVD	49.2	38.5	37
Respiratory	3.3	17.4	8
External	4.1	3.1	4

### Statistical analysis

Each daily mortality series was examined in relation to daily temperature using Poisson generalised linear models allowing for over-dispersion, following methods used in previous analyses for England and Wales [7]. Cubic smoothing splines of time with equally spaced knots were used to control for secular trends in the mortality series and any additional confounding by seasonally-varying factors other than temperature. The same level of seasonal control was used on each series with 7 degrees of freedom (df) per year, roughly equivalent to a two-month moving average, specified for the splines. We chose the number of df as a compromise between providing adequate control for unmeasured confounders and leaving sufficient information from which to estimate temperature effects. Sensitivity analyses were conducted to confirm that estimates were largely unchanged if other levels of seasonal control were considered.

Daily levels of PM_10 _and O_3 _(both average of the current and previous day) were incorporated into each regression model as possible confounding variables, regardless of statistical significance. Indicator variables for day-of week and public holidays were included. As cold effects can be apparent during months of even moderate temperatures, it was decided to have some degree of control for low temperatures. Cold effects are more delayed than for heat [18], and so cold effects were considered using a linear term of lag 0–13 (temperature averaged across values 0–13 days before the day of death) below a cut-point set at the 5th percentile of daily mean temperature.

To establish the general relationship between mortality and temperature, natural cubic splines of the temperature series (df = 3) were regressed against model residuals after controlling for the confounding factors noted above. The heat effect was modelled using lag0–1 (temperature averaged across values on the same day and the previous day). Heat effects were quantified assuming simple linear threshold models for each city. Thus, the heat effect is the log-linear increase in risk above a heat cut-point defined as the 95th percentile of daily mean temperature (lag 0–1) (Table 1). All analyses were conducted using STATA v9 [19].

## Results

Table 1 describes the characteristics of the mortality and environmental datasets in each of the three cities. The crude mortality rate in Budapest (4 per 1000 people in population) was high compared with London and Milan (2 per 1000), although the study periods were slightly different. Milan has a more elderly population compared to the other two cities. Summertime average of daily mean temperature was highest in Milan. Budapest and Milan have greater inter-annual temperature ranges because of their continental location. The "heat cut-points" (°C) used in this analysis were 24.4, 20.4, and 26.3, in Budapest, London and Milan, respectively. Observed PM_10 _and ozone levels were highest in Budapest and Milan.

**Figure 1:**
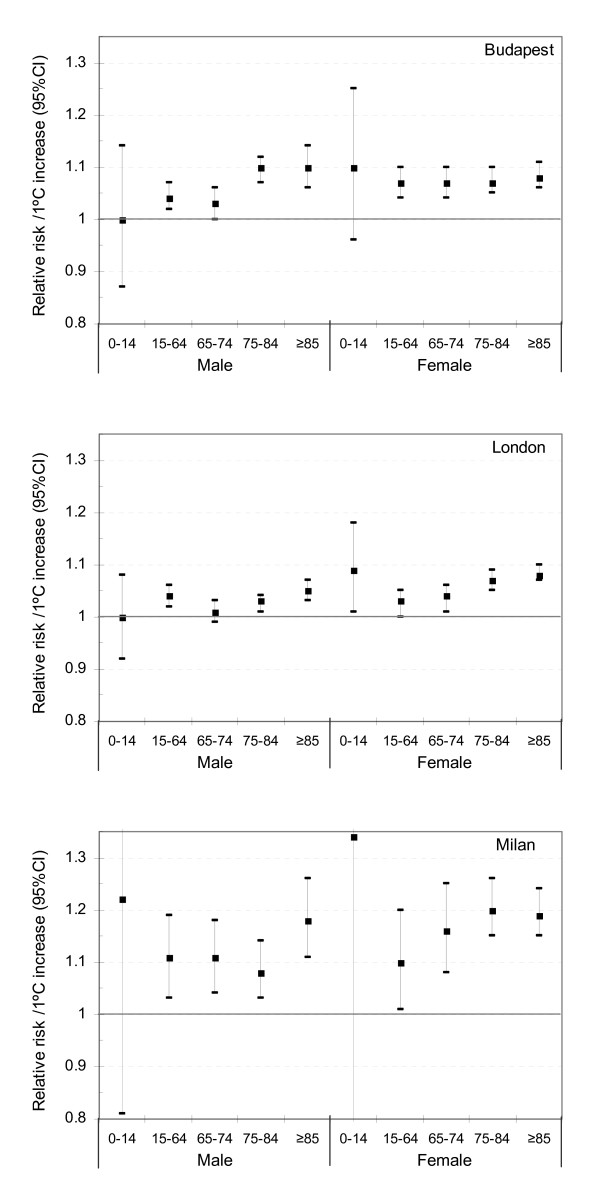
Relative risk of heat-related death for every 1°C change above the each cut-point.

The risk of heat-related mortality varied by age, sex, and cause of death, but with similar patterns in all cities. Figure 1 shows the relative risk (RR) of all-cause mortality by sex and age group. Heat effects are in general greater as age increases, and greater in females than in males, but there was some heterogeneity between the cities. Risk of death increased more steeply above the heat cut-point for both 75–84 and ≥85 years compared to younger age groups in all cities. The effect of heat on mortality in children was not statistically significant (except in females in London), and such estimates were unstable due to the very small numbers of deaths in this age group.

**Figure 2:**
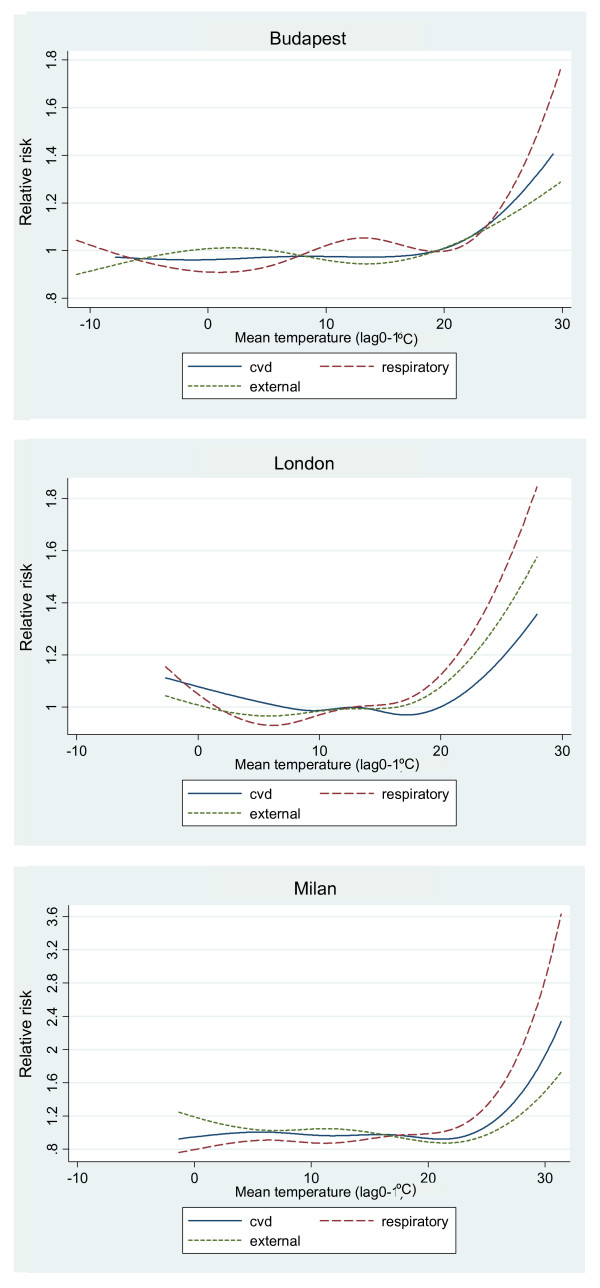
Adjusted relationship between relative risk of death and mean temperature by cause of death.

Deaths from respiratory causes were most sensitive to high temperatures (Figure 2). Effects of high temperature on death from external causes were apparent in all cities, although not statistically significant in Budapest. The risk was highest for the elderly age-group in London, whereas the non-elderly appeared to be at greatest risk from heat-related mortality from accidents and injuries in Budapest and Milan.

Table 2 estimates heat effects by cause of death and deprivation separately for the elderly (≥75) and the non-elderly (<75) age groups. There was no clear gradient in heat-related mortality across the deprivation quintiles in Budapest and London. A statistically significantly *decreasing *gradient in risk was observed with increasing deprivation level in Milan only for the non-elderly (<75), but not in the elderly age group.

**Table 2:** Percent change in deaths (95%CI) per °C increase in temperature above cut-point

	Budapest		London		Milan	
	
	Aged <75 year	Aged ≥ 75 year	Aged <75 year	Aged ≥ 75 year	Aged <75 year	Aged ≥ 75 year
Cause of death						

All cause	1.03 (1.02 to 1.05)	1.06 (1.04 to 1.07)	1.03 (1.02 to 1.04)	1.06 (1.05 to 1.07)	1.12 (1.08 to 1.16)	1.17 (1.14 to 1.20)
CVD	1.04 (0.99 to 1.08)	1.08 (1.04 to 1.12)	1.03 (1.01 to 1.04)	1.06 (1.05 to 1.07)	1.15 (1.07 to 1.24)	1.20 (1.15 to 1.25)
Respiratory	1.06 (0.98 to 1.14)	1.08 (1.01 to 1.15)	1.05 (1.01 to 1.08)	1.08 (1.06 to 1.10)	1.37 (1.15 to 1.62)	1.22 (1.14 to 1.31)
External	1.04 (0.98 to 1.11)	1.02 (0.96 to 1.09)	1.06 (1.02 to 1.10)	1.10 (1.02 to 1.18)	1.21 (1.05 to 1.40)	1.18 (1.03 to 1.34)

Deprivation						

1 (least deprived)	1.03 (1.01 to 1.06)	1.06 (1.03 to 1.09)	1.03 (1.01 to 1.05)	1.06 (1.04 to 1.08)	1.19 (1.11 to 1.27)	1.21 (1.16 to 1.26)
2	1.03 (1.01 to 1.06)	1.04 (1.01 to 1.07)	1.04 (1.02 to 1.06)	1.05 (1.04 to 1.07)	1.13 (1.05 to 1.22)	1.13 (1.08 to 1.20)
3	1.02 (0.99 to 1.06)	1.06 (1.03 to 1.10)	1.02 (0.99 to 1.04)	1.07 (1.05 to 1.09)	1.11 (1.02 to 1.20)	1.11 (1.05 to 1.17)
4	1.04 (1.02 to 1.07)	1.06 (1.03 to 1.08)	1.03 (1.01 to 1.05)	1.07 (1.05 to 1.09)	1.09 (1.00 to 1.19)	1.20 (1.13 to 1.27)
5 (most deprived)	1.03 (1.01 to 1.06)	1.07 (1.04 to 1.10)	1.02 (1.00 to 1.05)	1.04 (1.03 to 1.06)	1.00 (0.90 to 1.12)	1.18 (1.10 to 1.26)

There was little evidence in Budapest and London of modification of heat effects by quintiles of the proportion of people living in flats and the proportion of elderly people (≥65 years) living alone (data not shown). These variables were not available for Milan.

## Discussion

We used a standard approach to investigate heat effects in the three European cities and observed broadly consistent patterns in different sub-groups. Our results confirm that elderly people are most at risk from heat-related mortality, and, within this group women seem to be at increased risk compared to men in London and Milan.

When analyzing by cause, we observed the strongest heat effect on deaths from respiratory and cardiovascular diseases, as reported by other studies [20-23]. This is important from a public health point of view since cardiovascular disease is the leading single cause of death in Europe: 49% of all deaths in Budapest, 39% in London, and 37% in Milan during the study period. Most previous studies of weather and health have focussed on cardio-respiratory causes of death. However, our results suggest that mortality from external causes is also sensitive to heat, and that this effect is apparent in both adults and the elderly. A previous study in England and Wales, also found a heat signal in deaths from external causes [7], and indicated a stronger heat effect in the 0–64 years age group.

The exact mechanism by which heat affects mortality from external causes needs further investigation. The risk of accidents from traffic accidents, falls, and drowning are known to be affected by summer weather [24-27]. Cause of death certification in the elderly is often not accurate and a proportion of the deaths from cardiovascular and respiratory disease are likely to be misclassified[28]. Deaths from external causes, however, are unlikely to be misclassified. Although heat stroke deaths are underreported [29], deaths certified as due to classical heat illness are unlikely to contribute significantly to the number of deaths from external causes in our three cities.

We found little evidence of heat effects on mortality in children in Milan and Budapest, although there was some indication of a small effect on mortality in girls in London. A previous study in Oslo reported no effect of temperature on child mortality [21]. Overall our study indicates that heat does not have a significant effect on mortality in children, despite their limited ability to thermoregulate. There is no clear physiological mechanism to explain a gender difference for heat effects in children, although some studies have shown gender differences in physiological heat responses in adults [30]. More research is needed on the effects of heat on mortality and morbidity in children.

In Europe, epidemiological studies have shown little or no effect of socio-economic deprivation in the risk of heat-related mortality. Our results were broadly consistent with these reports, showing no clear gradient in heat-related mortality across the deprivation quintiles in the three cities except for the non-elderly population in Milan. It is possible that analyses of district and small area indicators are not able to detect a true but small effect on risk. In the UK, the Office for National Statistics has developed the MDI by Super Output Area (SOA) which has homogeneous characteristics (average population per SOA = 1500) from the 2001 census. There is no such published index for socio-economic deprivation in Hungary and Italy. For Budapest, we used the percentage of people who completed at least secondary school as it was more likely to have a normal distribution with a wider range than other possible indicators available in the census 2001. Area-level markers were used to represent individual circumstances and are thus subject to the ecological fallacy. Each unit area contains 32,534 households in Budapest, 4,765 households in London, and 118 households in Milan.

This study is not comparing estimated heat effects across cities, but only across subgroups within each city. There are important differences in sensitivity to heat between cities determined by weather other than temperature, air pollution, and other factors, such as population characteristics, cultural behaviours, and housing. The estimates of heat effects for subgroups in Milan appear large compared to the corresponding subgroups in the other cities. One reason is that the 95th percentile cut-point (26.3°C) in this study is rather high compared to a previous study in Milan [31] which suggested a cut-point of 23°C determined by model fit using data for 18 years (1985–2002). Applying a cut-point of 26.3°C results implies that only the more extreme hot days contribute to the mortality risk. Nevertheless the focus of the current study was on comparisons within cities and not between.

## Conclusion

Our study provides consistent evidence of the environment and social determinants of heat-related mortality in three European cities. Further research should focus on identifying the spatial distribution of heat-related mortality, particularly in relation to heat island "hot spots" and housing characteristics.

## Competing interests

The author(s) declare that they have no competing interests.

## Authors' contributions

AI, SH, RSK and LB conceived the study and participated in its design and coordination. AI, SH, MR and AR performed the statistical analysis. AI drafted the manuscript with contributions from all other authors.
